# Effect of crown-root ratio in endo-perio treated teeth restored with prosthodontic crowns

**DOI:** 10.6026/973206300220625

**Published:** 2026-02-28

**Authors:** Suruchi Sisodia, Ruchi patel, Kashish Bhawar, Deepak Kumar, Gatha Mohanty, Bhoomi Shah

**Affiliations:** 1 Department of Conservative Dentistry and Endodontics, Modern Dental College and Research Centre, Indore, Madhya Pradesh, India; 2 Department of Periodontology and Oral Implantology, Dasmesh Institute of Research And Dental Sciences, Faridkot, Punjab, India; 3 Department of Periodontics & Oral Implantology, Institute of Dental Sciences, Siksha 'O' Anusandhan University, Deemed to be University, Bhubaneswar, Odisha, India; 4 Department of Dentistry, College of dental science and research center, Ahmedabad, Gujarat, India

**Keywords:** Crown-root ratio, endo-perio lesion, prosthodontic crown, tooth survival, bone loss

## Abstract

The crown-root ratio (CRR) plays a crucial role in determining the biomechanical stability and long-term prognosis of endo-perio treated teeth restored with prosthodontic crowns. Therefore, it is of interest to assess the influence of CRR on the clinical performance and survival of such teeth in a sample of 60 patients aged 25-65 years. Based on radiographic CRR, teeth were categorized into three groups: favourable (≤1:1), moderate (1:1-1.5:1) and unfavourable (>1.5:1). All patients received standardized endodontic, periodontal and prosthodontic treatment and were evaluated over a 12-month period for probing depth, attachment level, marginal bone changes and crown survival. Teeth with favourable CRR showed minimal bone loss (0.2 mm) and a 100% survival rate, whereas those with unfavourable ratios demonstrated increased bone loss (0.9 mm) and reduced survival (75%) (p < 0.05). Thus, we show that prognosis declines progressively as CRR becomes less favourable, highlighting the importance of radiographic CRR assessment in treatment planning for enhancing the long-term success of prosthodontically restored endo-perio treated teeth.

## Background:

The restoration of endodontically treated teeth represents a critical challenge in prosthodontics, as these teeth are structurally compromised and more prone to fracture under functional loads. Several studies have emphasized that the longevity of restorations is influenced by both the amount of remaining tooth structure and the type of restorative materials used. The preservation of coronal and radicular dentin is essential to maintain the biomechanical integrity of the tooth and to minimize the risk of fracture during function [1]. Clinical evidence indicates that teeth restored with posts and cores exhibit varying survival rates depending on the post system, the type of core material, and the coverage provided by the final restoration [2]. The mechanical behavior of these restorations is further influenced by the loading patterns encountered during mastication and parafunctional habits, which can exacerbate stress concentration in endodontically treated teeth [3]. Endodontically treated teeth also show reduced fracture resistance due to the loss of structural integrity from caries, access cavity preparation, and previous restorations [4]. Studies suggest that the use of adhesive systems and fiber-reinforced posts can enhance the distribution of occlusal forces, thereby improving the overall fracture resistance of these teeth [5]. Moreover, proper selection of restorative materials, such as high-strength ceramics or composite cores, can contribute to long-term success and prevent catastrophic failures [6]. Given the complex interplay of mechanical and biological factors, understanding the fracture resistance and survival outcomes of different restorative approaches remains a priority for clinicians. Therefore, it is of interest to examine recent evidence regarding the performance of zirconia-based prostheses, fiber posts and endodontically treated teeth to provide insights into optimal restorative strategies.

## Methodology:

A prospective clinical observational study was performed to evaluate the impact of the crown-root ratio (CRR) on the prognosis of endo-perio treated teeth restored with prosthodontic crowns. The research was conducted in the Department of Prosthodontics and Periodontology at a tertiary dental care institution over duration of twelve months. A total of sixty patients, aged 25 to 65 years, necessitating prosthodontic rehabilitation of endodontically and periodontally treated teeth, were included following comprehensive clinical and radiographic assessment. Patients were chosen based on particular inclusion and exclusion criteria. Patients were eligible if they had successfully completed endodontic and periodontal therapy, had teeth that were suitable for full-coverage prosthodontic crowns and had at least one-third of the root embedded in alveolar bone with adequate coronal tooth structure available for restoration. The exclusion criteria encompassed teeth exhibiting root fractures or perforations, patients with uncontrolled systemic diseases such as diabetes or hypertension, teeth demonstrating severe mobility (Grade III) and individuals unwilling to attend scheduled follow-up visits. The chosen teeth were categorised according to their radiographically assessed crown-root ratios, employing the paralleling technique for periapical radiographs. Group I had teeth with a good CRR (≤1:1), Group II had teeth with a moderate CRR (between 1:1 and 1.5:1) and Group III had teeth with a bad CRR (>1.5:1). All teeth were treated according to standard procedures, which included checking the quality of endodontic obturation, periodontal therapy like scaling; root planing and regenerative procedures when needed and then preparing the teeth for full-coverage restorations. Metal-ceramic crowns were made and then cemented with resin-modified glass ionomer cement while being properly isolated. We looked at the restoration again at baseline (right after cementation), 6 months and 12 months. The clinical and radiographic parameters evaluated encompassed tooth mobility (utilising Miller's Index), periodontal probing depth (PPD), clinical attachment level (CAL), marginal bone level, crown debonding or failure and overall tooth survival. We used SPSS software version 26.0 to do a statistical analysis of the data we had collected. We used descriptive statistics (mean ± standard deviation) to look at all of the continuous variables. We used the Chi-square test for categorical data and one-way ANOVA for continuous data to look at the relationship between crown-root ratio and clinical prognosis. A p-value below 0.05 was deemed statistically significant.

## Results:

**Figure 1:**
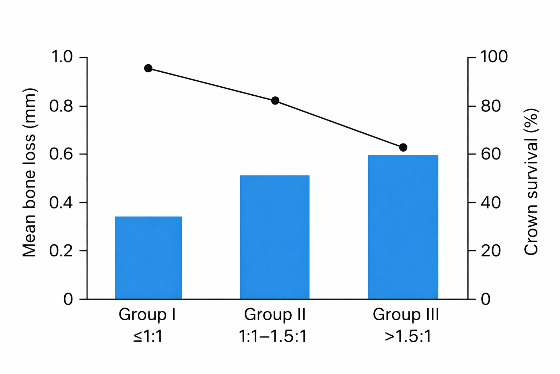
Relationship between crown-root ratio, mean bone loss, and crown survival among study groups

Sixty patients took part in the study and each one brought in one endo-perio treated tooth that had been restored with a full-coverage prosthodontic crown. There were 20 teeth in Group I (favourable CRR ≤1:1), 20 teeth in Group II (moderate CRR between 1:1 and 1.5:1) and 20 teeth in Group III (unfavourable CRR >1.5:1). All participants finished the follow-up period of 12 months. At the start, all groups had good periodontal health and enough functional stability. Over the course of 12 months, Group I had the best results, with only small changes in probing depth, attachment loss, or marginal bone resorption. Group II showed moderate changes, with slightly more mobility and probing depths. Group III, on the other hand, showed a clear drop in periodontal stability, more tooth mobility and a higher rate of crown debonding and bone loss. In Group I, the mean periodontal probing depth (PPD) went from 2.1 mm at the start to 2.4 mm after 12 months. In Group II, it went from 2.3 mm to 3.1 mm and in Group III, it went from 2.5 mm to 3.8 mm. The mean clinical attachment level (CAL) loss was also small in Group I (0.3 mm), moderate in Group II (0.7 mm) and large in Group III (1.2 mm). The average loss of marginal bone was 0.2 mm for Group I, 0.5 mm for Group II and 0.9 mm for Group III at the end of the study period. In Group I, 100% of prosthodontic crowns survived after 12 months. In Group II, 90% survived and in Group III, 75% survived. One-way ANOVA and Chi-square tests showed that there was a strong link between the crown-root ratio and both tooth mobility and bone loss (p<0.05). These results indicate that a less favourable crown-root ratio correlates with an elevated risk of mechanical and biological complications, resulting in a deterioration of the long-term prognosis and survival of the restored teeth. Table 1 shows that the mean probing depth, attachment loss and bone resorption all went up as the crown-root ratio went up. Figure 1 shows that there is an inverse relationship between bone loss and crown survival. This means that teeth with an unfavourable CRR (>1.5:1) lost more bone and had lower survival rates than teeth with a favourable CRR.

## Discussion:

The evaluation of zirconia-based single crowns and fixed dental prostheses has demonstrated excellent long-term clinical performance. A prospective observational study reported a fifteen-year recall period on zirconia-based restorations, revealing high survival rates and minimal technical complications [15]. This highlights the material's capacity to withstand functional loads and its favorable mechanical properties, including fracture toughness, wear resistance, and high flexural strength. These characteristics make zirconia a preferred material in both single crowns and fixed partial dentures, particularly in patients with high masticatory forces or parafunctional habits. Moreover, the use of monolithic zirconia reduces the risk of veneering ceramic chipping, which has historically been a common failure mode in bilayered ceramic restorations. Survival and success rates of restorations post-endodontic treatment vary significantly depending on the restorative approach. Hayati *et al.* observed that endodontically treated teeth restored with direct or indirect restorations demonstrated survival rates exceeding 80% over a five-year period, with failures largely associated with inadequate coronal coverage, excessive tooth structure loss, or suboptimal restorative design [16]. This underscores the importance of proper treatment planning, as restorations must not only replace lost tissue but also reinforce the tooth structure to resist functional loads. The choice of material, post design, and adhesive technique all contribute to long-term stability, as these factors directly influence stress distribution within the tooth. The long-term survival of anterior teeth restored with fiber posts has also been extensively investigated. Signore *et al.* reported that maxillary anterior teeth restored with either tapered or parallel-sided glass-fiber posts combined with full-ceramic crowns maintained favorable survival rates over multiple years, suggesting that post design influences stress distribution but does not significantly affect overall longevity when proper coronal coverage is provided [17]. These findings indicate that fiber posts are particularly suitable for esthetic zones where preservation of tooth structure and minimal invasiveness are critical. Fiber posts also offer the advantage of a modulus of elasticity similar to dentin, which allows more even force distribution and reduces the likelihood of catastrophic root fractures. Mechanical strength of zirconia fixed partial dentures has been systematically reviewed, revealing that connector dimensions, pontic design, and occlusal adjustments critically impact fracture resistance [18]. The review emphasized the role of functional load simulations in laboratory studies, which demonstrate that even minor variations in connector cross-section or pontic thickness can significantly affect the probability of fracture under cyclic loading. Appropriate prosthetic design, including optimal connector dimensions and occlusal alignment, can mitigate common failure modes such as connector fracture or veneering ceramic chipping. This evidence highlights that clinical success depends not only on the material's inherent strength but also on careful prosthetic planning and design optimization. In addition, fracture resistance studies on endodontically treated teeth restored with fiber posts or zirconia crowns have shown that the selection of adhesive systems and cementation protocols can substantially affect outcomes. Özyürek *et al.* demonstrated that teeth restored with fiber posts exhibited higher fracture resistance when bonded with dual-cure resin cements compared to conventional luting techniques [7]. Unfavorable crown-root ratio significantly compromises the biomechanical stability and long-term prognosis of restored teeth, particularly in cases with reduced periodontal support [8]. This finding underscores the critical role of adhesion in reinforcing the tooth-restoration complex, as well as the importance of selecting materials that provide both mechanical and chemical bonding to dentin. Sorrentino *et al.* evaluated the mechanical performance of zirconia-based fixed partial dentures, finding that fatigue testing simulating masticatory forces led to predictable fracture patterns and validated the suitability of zirconia for long-term restorations [14]. Similarly, Khijmatgar *et al.* highlighted the low incidence of chipping or connector failure in single crowns and FPDs over a fifteen-year period, reinforcing the reliability of monolithic zirconia even under high functional demand [15]. These findings collectively demonstrate that zirconia's favorable mechanical properties and clinical predictability make it an ideal material for both anterior and posterior restorations. Patil *et al.* focused on the influence of post design and material on fracture behavior, showing that endodontically treated teeth restored with tapered fiber posts were less prone to catastrophic fractures than those restored with metallic posts, due to improved stress distribution and better adaptation to the root canal anatomy [9]. Fennis *et al.* further demonstrated that posterior teeth restored with fiber posts and adhesive core materials exhibit higher fatigue resistance compared to conventional post and core systems [11]. These results highlight the importance of post selection, emphasizing that fiber posts reduce the risk of root fractures while allowing effective restoration of structural integrity. Ng *et al.* conducted a systematic review analyzing outcomes of endodontic treatment and subsequent restorations, reporting that teeth with adequate coronal coverage had significantly higher survival rates, regardless of post placement [10]. This finding reinforces the principle that preserving remaining tooth structure and providing full coronal coverage are paramount for long-term success. Zicari *et al.* similarly emphasized the role of full-coverage restorations in preventing fractures and ensuring longevity of endodontically treated teeth [12]. Together, these studies confirm that restorative strategies should prioritize both structural preservation and appropriate coverage to achieve optimal outcomes. Linn and Messer highlighted that fracture resistance is directly correlated with remaining tooth structure and proper restoration technique, and that even high-strength posts cannot compensate for extensive dentin loss [13]. Signore *et al.* reinforced that fiber posts, when combined with ceramic crowns, provide predictable survival, particularly in the esthetic zone, due to their favorable mechanical compatibility with dentin [17]. Finally, Hayati *et al.* and Gargari *et al.* together underscore that both material selection and prosthetic design are critical for long-term success. Fiber-reinforced posts and monolithic zirconia restorations offer superior performance due to their favorable mechanical properties, while careful occlusal management and adhesive protocols further enhance restoration longevity [16, 18]. Overall, the evidence suggests that endodontically treated teeth benefit from restorations that optimize stress distribution, preserve remaining tooth structure, and utilize high-strength, durable materials. Zirconia crowns and FPDs, fiber posts, and proper adhesive techniques collectively contribute to long-term survival and resistance to fracture, providing a reliable and predictable restorative solution for structurally compromised teeth. The integration of appropriate material selection, post design, and prosthetic planning ensures both functional and esthetic success over extended periods, reinforcing the importance of comprehensive restorative strategies in modern prosthodontics.

## Conclusion:

It can be inferred that the crown-root ratio is a significant factor influencing the prognosis of endo-perio treated teeth restored with prosthodontic crowns. Teeth exhibiting a favourable CRR (≤1:1) demonstrated enhanced clinical performance, negligible bone loss and elevated survival rates in contrast to those with moderate or unfavourable ratios. As the CRR gets worse, the biomechanical load on the tooth and the structures that support it gets heavier. This makes the tooth less stable and more likely to fail. So, a careful radiographic evaluation of the CRR should be a key part of planning for diagnosis and treatment to make sure that prosthodontic rehabilitation works as well as possible for both function and biology.

## Figures and Tables

**Table 1:** Comparison of clinical parameters among different CRR Groups

**Parameter**	**Group I (≤1:1)**	**Group II (1:1-1.5:1)**	**Group III (>1.5:1)**	**p-value**
Mean PPD (mm)	2.4 ± 0.3	3.1 ± 0.4	3.8 ± 0.5	<0.05*
Mean CAL Loss (mm)	0.3 ± 0.1	0.7 ± 0.2	1.2 ± 0.3	<0.05*
Marginal Bone Loss (mm)	0.2 ± 0.1	0.5 ± 0.2	0.9 ± 0.3	<0.05*
Crown Survival Rate (%)	100	90	75	<0.05*
*Statistically significant
